# Exploring the Frontiers of Technology in Forensic Psychiatric Youth Care: Ethical Challenges

**DOI:** 10.1007/s10802-025-01358-2

**Published:** 2025-09-06

**Authors:** Hanneke Kip, Hanneke Scholten, Gerben Meynen

**Affiliations:** 1 Centre for eHealth & Wellbeing Research, Department of Human & Institutional Behaviour, University of Twente, Enschede, The Netherlands; 2 Department of Research, Transfore, Deventer, The Netherlands; 3 Department of Child and Adolescent Psychiatry/Psychology, Erasmus MC, Rotterdam, The Netherlands; 4 Willem Pompe Instituut Voor Strafrechtswetenschappen, Utrecht University, Utrecht, The Netherlands; 5 Department of Philosophy, VU University of Amsterdam, Amsterdam, The Netherlands; 6 Utrecht Centre for Accountability and Liability Law (UCALL), Utrecht, The Netherlands

**Keywords:** Forensic youth psychiatry, Forensic psychiatry, Ethics, Technology, eHealth interventions, Digital health

## Abstract

Technologies such as virtual reality, wearables, and mobile apps have the potential to improve forensic psychiatric treatment of youths. Meanwhile, these technological advancements have given rise to new, complex ethical challenges. Paying attention to ethics is especially relevant in forensic psychiatric youth settings because of the often coercive context of treatment and the vulnerable patient population. The goal of this viewpoint paper is to identify and discuss important ethical challenges regarding the use of technology in forensic psychiatric youth care. In line with approaches within the domain of ethics, an analysis of relevant scholarly literature was used for this viewpoint paper. First, a general description, an overview of research on effectiveness, and examples from practice are provided for six technologies that can be used in forensic psychiatric youth care: virtual reality, internet-based interventions, mobile apps, wearables, neurotechnology, and games. Next, ethical challenges that are relevant for these technologies are explored, related to informed consent, privacy and data security, reliability and validity, equity, accessibility and usability, undesirable side effects, acceptability of content, persuasiveness, and evidence-based interventions. Interdisciplinary collaboration between researchers, patients, therapists, ethicists, technology developers, and forensic organizations is recommended for timely identification of ethical challenges and suitable solutions. We suggest that patients and therapists should be actively involved throughout all phases of the process, from development of the technology via co-creation to active participation in implementation and evaluation in practice.

## Introduction

Forensic psychiatry takes place at the intersection of law and mental healthcare and is focused on treatment of people who have committed an offense, which is at least in part caused by psychiatric disorders (Arboleda-Florez, 2006). Forensic psychiatric youth care is a specialized branch focused on treatment of justice-involved youths with psychiatric disorders, typically between the ages of 12 and 23. In general, treatment of forensic patients is challenging. Among other things, motivation for their—often obligatory—treatment is typically low (Drieschner et al., 2004; Teplin et al., 2013). Complex psychosocial problems and high levels of comorbidity further complicate treatment (Goethals et al., 2008). Additionally, many patients have low literacy and cognitive skills and have problems reflecting on their behaviour (Groen et al., 2011; Kip et al., 2020; Svensson et al., 2015). Despite these challenges, current treatment approaches, such as cognitive behavioural therapy (CBT), highly rely on talking and thinking about behaviour. However, such language-based and reflective treatments are challenging for many forensic patients because of the aforementioned cognitive, reflective and motivational problems. This can be especially difficult for youths. One of the reasons for this is that capacities such as planning, decision-making, and self-reflection are still developing. Consequently, in forensic psychiatric youth care, there is a need for innovative, experience-based approaches that rely less on language and cognitive reflection (Kip et al., 2019a, 2019b, 2019c).

Over the past few years, increasing attention has been given to the development and evaluation of eHealth technologies for forensic psychiatric settings by both researchers and forensic organizations. Systematic reviews on technology on treatment of both adults and youths who committed an offence have revealed a broad range of technologies that have been studied: videoconferencing, internet-based interventions, virtual reality (VR), social media, and games (Grove et al., 2021; Kip et al., 2018). A Dutch qualitative study has also shown that more technologies are being used in practice and research, such as wearables with biofeedback, mobile apps, and neurotechnology (Kip et al., 2019a, 2019b, 2019c). These reviews show that most research has focused predominantly on language-based technologies such as internet-based interventions, even though practitioners express a need for more experience-based approaches such as VR, wearables, or neurotechnology (Kip et al., 2019a, 2019b, 2019c). Indeed, while there is a need for more research, exploratory research shows that interactive virtual reality (VR) can, for example, support realistic roleplay in context, contributing to a better development and generalization of, e.g., coping skills (Kip et al., 2019a, 2019b, 2019c; Park et al., 2011). Furthermore, wearables with biofeedback or neurotechnology offer new ways to support forensic psychiatric patients in gaining more insight into changes in bodily processes, which can point to increasing stress or anger (Argese et al., 2020; ter Harmsel et al., 2023a, 2023b). Additionally, including game design principles in interventions can contribute to overcoming motivational issues, which can especially appeal to youths (Scholten & Granic, 2019). Furthermore, mobile apps can be used to deliver short and simple interventions to patients throughout the day, providing the opportunity to work on treatment objectives outside of appointments with therapists (Dekkers et al., 2023). However, despite the reported and potential advantages, the use of these new types of technology in forensic psychiatric youth care is still in its infancy.

One type of challenge that presents itself when these technologies are introduced in forensic psychiatric youth care is ethical in nature. While the use of technology in treatment of psychiatric patients often creates ethical challenges, forensic youth psychiatry has unique characteristics that give rise to specific concerns. These concerns have to do, among other things, with the coercive setting of forensic care, in which treatment is often an obligatory part of a sentence. This might cause patients to feel less free to refuse certain new interventions (Adshead & Davies, 2016; Szmukler & Appelbaum, 2008). Furthermore, when youths are treated, factors such as the role of parents and their consent for interventions become important, especially for underage patients (Schneble et al., 2021). Additionally, since the use of technology in forensic (youth) psychiatry is relatively new, not much is known yet about if, how, when, and for whom technologies should be used (Grove et al., 2021; Kip et al., 2018). In addition to practical questions related to, for example, integration into treatment protocols, indication criteria, and design challenges, there are also many unanswered and even unasked questions regarding the ethics of using technology in forensic treatment of youths.

The goal of this viewpoint paper is to identify and discuss several important ethical issues regarding the introduction and use of new technologies in forensic psychiatric youth care. In line with the characteristics of viewpoint papers in ethical research and due to the broad objective of this paper, this paper was created through a general examination of relevant literature and not through a systematic literature search.

The paper is structured as follows. First, we provide a brief overview of technologies that are used in forensic mental healthcare or research, focusing on their application for youths. We also pay attention to what is currently known and unknown about the effectiveness and application of these apps in a forensic context, as this is relevant from an ethical perspective as well. Next, we provide an overview of ethical issues related to several of these technologies. In the discussion, which we named the ‘ways forward’ section, we highlight future directions for research and practice.

## Opportunities of Technology for Forensic Psychiatric Youth Care

Ethical concerns are tied to the specific features of a technology. To understand these concerns, it is important to have knowledge about the possibilities and current state of affairs of technology in forensic (youth) psychiatry. In this section, technologies that are commonly studied or used in forensic settings are described: virtual reality, internet-based interventions, mobile apps, wearables with biofeedback, neurotechnology, and games. For each technology, we provide a general explanation, review its effectiveness in general and forensic psychiatry, and outline its application in youth forensic or other youth psychiatric settings. This overview is based on two systematic literature reviews on technology in treatment of juveniles (Grove et al., 2021) and of people who committed an offense (Kip et al., 2018), supplemented with Dutch qualitative studies with therapists, patients, and policy advisors (Bierbooms et al., 2015; Kip, Kip et al., 2019a, 2019b, 2019c). Because of the rapid pace of technological development, this overview is supplemented with a non-systematic search of scientific and grey literature on technology in psychiatry and similar settings.

### Virtual Reality

The term virtual reality (VR) refers to a group of technologies that are characterized by their ability to immerse people in digital environments that feel real and are thus able to elicit real emotions, often via head-mounted displays. In mental healthcare, different types of VR are used. A very rough distinction can be made between 360-degree VR and interactive VR. In 360-degree VR, the user is exposed to prerecorded videos such as calming natural environments or witnessing intimate partner violence through the eyes of a child. A second form is *interactive* VR, in which the virtual environment responds to the user. For example, patients and therapists can collaboratively build highly personalized virtual environments and engage in role-play. The therapist controls the environment via a dashboard and the voice of virtual avatars with a voice-morphing microphone.

Research on the use of VR in psychiatry in general, e.g., in treatment of anxiety, depression, and psychosis, has generally shown similar and, in some cases, even superior outcomes for VR compared with solely face-to-face therapy (Turner & Casey, 2014). A specific example of a VR application used in forensic psychiatry is Virtual Reality Aggression Prevention Therapy (VRAPT): a VR-training focused on practicing new behaviour in virtual roleplaying sessions, aiming to improve how a patient interprets and responds to social situations (Klein Tuente et al., 2020). An example is remaining calm during a conversation with a rude bouncer of a bar. A first RCT on VRAPT in Dutch forensic inpatient settings revealed small, short-term effects on secondary outcomes such as impulsiveness, but no impact on aggression was found, highlighting the need for further improvement of VRAPT and additional research (Klein Tuente et al., 2020). Another forensic example is the use of VR in the assessment of paedophilia. In research settings, people who committed an offense are exposed to avatars of naked children and adults, during which arousal is measured through penile plethysmography (PPG), a technique that measures blood flow to the penis via a ring (Trottier et al., 2019).

While little research has been conducted on the use of VR in treatment of forensic psychiatric youths, there are several examples. One application is Street Temptations, which is a 360-degree VR video in which the youths are immersed in a virtual street fight resulting from peer pressure. This scenario serves as a starting point for exercises that encourage the consideration of different perspectives and reflection on behaviour (Klein Schaarsberg et al., 2023). Another example is What’s up: an interactive VR application to assess youths who start treatment by assessing aggression via exposure to social triggers. More specifically, youths are placed on a virtual schoolyard, during which they are approached by peers in different ways in either friendly or unfriendly ways, during which time therapists score the response of the youth (Creemers et al., 2023).

### Internet-based Interventions

Internet-based interventions—sometimes also referred to as digital health interventions or online modules – are usually based on evidence-based treatment models such as CBT or mindfulness. Using a web-based approach, they present patients with knowledge and skills via multiple lessons that can be accessed via a PC or smartphone. The lessons contain, among other things, written information, reflective assignments, or multimedia applications such as videos (Andersson, 2018). While these interventions can be used independently by patients, they are often used as part of treatment: patients work on them on their own time, and therapists introduce and discuss them during sessions (Wentzel et al., 2016).

Research on internet-based interventions in mental healthcare in general has shown relatively positive results. Meta-analyses consistently show that internet-based interventions—especially those involving human support or those that are used in a blended way—are effective for a broad range of psychiatric disorders, such as depression or posttraumatic stress disorders, often resulting in outcomes similar to those of face-to-face interventions (Andersson et al., 2014; Andrews et al., 2018; Carlbring et al., 2018; Hedman‐Lagerlöf et al., 2023; Kuester et al., 2016; Păsărelu et al., 2017). However, these positive findings cannot simply be generalized to forensic mental healthcare (Kip et al., 2018). In a recent mixed-methods pilot RCT on an internet-based intervention on aggression, it appeared to be very difficult to include participants because of the low motivation of both therapists and patients to use the intervention and experienced mismatch between patients’ reading skills and the amount of written text (Klein Haneveld et al., 2024). Another forensic example is the Journey to Change program: a treatment adjunct that consists of three computer-administered sessions and an accompanying print guide (Levesque et al., 2012). A pilot study showed promising outcomes, e.g., on readiness to change and physical violence. Even though these types of interventions were introduced in forensic practice more than 15 years ago, little is known about their effectiveness due to the low number of studies and high heterogeneity of populations (Kip et al., 2018). Implementation research has shown that internet-based interventions are generally not used much in forensic outpatient care, despite the intentions of organizations (Kip et al., 2020).

A review on the use of technology in assessment and treatment of justice-involved youths identified multiple internet-based interventions, such as online CBT programs, focused on, e.g., emotion regulation, interpersonal functioning, mindfulness skills, or sexual health (Grove et al., 2021). Not enough studies were presented to draw conclusions about effectiveness, but most studies reported outcomes of internet-based interventions that were at least similar to those of regular treatment (Grove et al., 2021; Kip et al., 2018). A specific example is SPARX-, which uses a virtual therapist and play-based exploratory learning in a game-like environment to teach and rehearse skills, e.g., relaxation, problem solving, emotion regulation, and improved social skills (Fleming et al., 2019). As is the case in more studies on internet-based interventions in forensic settings, usage was too low to assess effectiveness.

### Mobile Apps

Mobile apps refer to applications that can be downloaded on a smartphone and/or tablet and are thus always accessible by the user. While there are some similarities with internet-based interventions—especially compared to somewhat older apps—there are also many differences between their affordances. For example, apps offer more opportunities in terms of adding persuasive features such as monitoring, reminders, and personalized coaching. An important difference is that—as opposed to laptops or computers – smartphones are almost always close to the user, which means that there are more opportunities to offer treatment and collect information throughout the user’s daily life. Generally, most apps have a single objective, e.g., providing mindfulness exercises, providing a broad range of tools for dealing with ADHD, or keeping a diary on depressive complaints.

Research on mental health apps has shown results comparable to those of more extensively studied technologies, such as internet-based interventions. In general, they are viewed as promising in treatment of psychiatric disorders such as depression and anxiety disorders (Mahreen et al., 2024). In forensic mental healthcare, few studies on apps have been conducted. A recent review on apps and other phone-based interventions identified only 15 studies, of which only 2 assessed the effectiveness of a mobile application (Aarts et al., 2024). An example of an app focused on people convicted of child sexual abuse is Troubled Desire, which contains self-assessment items on sexual interests and sexual behaviour (Schuler et al., 2021). Other apps focus on support during probation, e.g., by providing didactive modules on, for example, stress reduction and daily monitoring of risk factors (Carswell et al., 2022). Another example is the HKT-spider, an app that can be individually used by inpatients to assess themselves via a risk assessment instrument that is usually only filled out by professionals to facilitate shared decision-making (Horst et al., 2023).

The aforementioned review identified only two studies that focused on the use of apps in forensic youth care (Grove et al., 2021). An example is a feasibility study on a daily monitoring app for justice-involved youths with a mild intellectual disability, asking about, e.g., mood, anxiety, and impulsivity. The study revealed generally high acceptability, with high compliance rates in outpatient and residential care settings but not in juvenile detention settings (Hulsmans et al., 2023). Another diary app is currently being studied in forensic psychiatric youth care, but instead of text, more emphasis is placed on emojis to better tailor the design to the preferences of youths (Leijse et al., 2024). A feasibility study showed the potential to increase understanding of emotions and behaviours, but the app did not seem to contribute to treatment engagement (Leijse et al., 2024). A scoping review of mHealth apps for youths in general identified only a small number of studies, and suggested limited but positive evidence for the effectiveness of these apps in improving mental health outcomes (Litke et al., 2023).

### Wearables with Biofeedback

Wearables are electronic devices that can be worn on the body and collect various types of data through sensors. Wearables can be designed in many different shapes and sizes, such as simple wristbands, watches, chest straps, rings, patches, or clothing items such as socks and shirts (Cornet et al., 2017). Data can be related to physical and mental health and can be used to develop a composite score or digital biomarker, such as stress or anger (Jarczok et al., 2022). Some wearables only collect data without providing direct feedback to the user, such as the research tool Empatica E4. However, in most cases, there is some form of biofeedback. In this case, the user receives real-time information about a biometric value or its changes, allowing them to independently monitor aspects such as heart rate or sleep. This can happen directly through the wearable itself—think of a watch that vibrates when the heart rate exceeds a certain threshold—or through a corresponding mobile app. Depending on the type of wearable device, it may not only monitor physiological variables but also provide"coaching"by offering a (small) intervention, such as a breathing exercise.

A systematic review examined biofeedback through the use of wearables for emotion regulation in general (ter Harmsel et al., 2021). Most of the included studies reported that the use of wearables had a positive effect on stress. However, due to the low number of studies, no conclusions could be drawn regarding effectiveness for psychiatric populations, and even less is known about forensic psychiatry. An example of a wearable that was specifically designed for psychiatric patients and was recently studied in a forensic setting, is the Sense-IT (Derks et al., 2019). The app provides notifications of increased heartrates and provides suggestions to users, e.g., to notice their breathing. In a recent study with 25 forensic psychiatric patients, some, but not all, clients experienced positive effects from the wearable (ter Harmsel et al., 2023a, 2023b). Another technology specifically developed for forensic patients is the GRIP app, which uses a chest strap and an accompanying app with small coaching interventions (Argese et al., 2020). In addition, commercial wearables are sometimes used in practice as well (Kip et al., 2019a, 2019b, 2019c). These wearables, such as the Apple Watch, Garmin Vivosmart, or the Samsung Watch, are often focused on general fitness and collect data related to various lifestyle aspects, such as exercise, sleep, stress, and physical (in)activity (Kip et al., 2019a, 2019b, 2019c). A study on the use of these wearables in Dutch forensic outpatient care reported similar results as those of the Sense-IT: for some patients, the wearable approach seemed to be effective in increasing interoceptive awareness, but for others, it was not effective, (Heirbaut et al., 2024). This highlights the importance of the ‘what works for whom’ question, since wearables do not seem to be effective for all patients. Furthermore, all studies emphasize the difficulty of implementing wearables in forensic practice.

A review on technology in treatment of forensic youth did not identify any studies on the use of wearables or biofeedback (Grove et al., 2021). Nevertheless, on the basis of research thus far and experiences from practice, wearables seem to be suitable for youths as well, among other things, because many youths might already be familiar with fitness watches, lowering the threshold to use them.

### Neurotechnology

Neurotechnology refers to devices and methods for monitoring and influencing brain function. In other words, some neurotechnologies can obtain *information* from the brain, and others can *alter* the brain. Neuroimaging techniques such as magnetic resonance imaging (MRI) or functional MRI (fMRI) belong to the first category and allow clinicians and researchers to visualize and analyse brain correlates of disorders or aggressive behaviour. More specifically, it can help identify the presence, for example, of traumatic brain injury (TBI) or other types of neurological pathology, such as stroke and tumours, that might explain transgressive behaviours. In some court cases, neuroimaging is also used in forensic assessments of people who committed offenses (De Kogel & Westgeest, 2015; van Dongen et al., 2024). In the second case, neurotechnology is used to intervene in the brain, which can potentially influence mood, behaviour, or specific cognitive functions. Examples include deep brain stimulation (DBS), transcranial magnetic stimulation (TMS) or transcranial direct current stimulation (tDCS).

An application of neurotechnology that is specific for forensic psychiatry is its addition to risk assessment. While there is a need for more and better research, there are indications that brain scans may contribute to better risk assessment of forensic patients in the future (van Dongen et al., 2024). For example, researchers have studied a sample of released persons and found that they could predict who would be rearrested in a 4-year period on the basis of fMRI data obtained during the performance of an impulse-control task (Aharoni et al., 2013). Neuroimaging data was also included alongside traditional risk factors, and adding brain scan data improved the prediction of recidivism in psychiatric patients (Delfin et al., 2019). Another possible way of using neurotechnology in forensic psychiatry is neurofeedback. In this intervention, signals from the brain are monitored and then shown to patients, who are asked to control these signals by applying self-regulation techniques such as deep breathing. In one study, forensic patients learned to control EEG activity via a gamified, computerized task, leading to reduced reported craving, but in a small percentage of participants due to low feasibility (Fielenbach et al., 2019). tDCS is a neuromodulation technique that delivers low-intensity, direct electrical currents via scalp electrodes (Nitsche & Paulus, 2000). Research indicates that tDCS can be an effective intervention for several psychiatric disorders, including schizophrenia, major depression, and addiction (Kuo et al., 2017; Trojak et al., 2017) and might also offer a possible intervention option for aggression (Sergiou et al., 2020). A Dutch study reported that tDCS in a forensic population resulted in reduced self-reported aggression (Sergiou et al., 2022). Finally, regarding the use of DBS in people who committed offenses, a German research group has hypothesized that this type of neuromodulation (requiring surgical implantation of electrodes in the brain) could be used to reduce sexual drive in people committed sexual offenses (Fuss et al., 2015).

Little is known about the use of neurotechnology in forensic youth psychiatry. In a sample of justice-involved young adults, adding biological data contributed to the prediction of recidivism (they included, among other data, EEG and baseline heart rate) (Zijlmans et al., 2021). Research in youth with mental illness in general has shown mixed effects. For example, an RCT on the use of neurofeedback as an addition to treatment of adolescents with ADHD revealed that behavioural problems decreased equally in both groups, showing that neurofeedback as an addition was not more effective than treatment as usual (Bink et al., 2015). Other non-experimental studies have shown promising results for the use of neurofeedback in adolescents with, for example, depression (Whitehead et al., 2022).

### Games

Multiple terms, such as serious games, applied games, or gamification, are used for the use of gaming elements in interventions. Although there are considerable differences between these terms (Becker, 2021), we discuss all of them here and refer to them with the umbrella term ‘games’. These types of games are designed with a primary purpose that is not entertainment. The underlying assumption is that by gamifying interventions, patients will be more engaged and adherent and thus benefit more from these interventions. A specific advantage for youths is that games align with their often existing interest in video games. If well designed, games can be intrinsically motivating, offer a strong sense of agency, and are simply fun (Granic et al., 2014). Compared with the previously described categories, games are not a specific type of technology but refer to a *way of delivering* interventions. Games can be accessed by users via a broad range of technologies, such as mobile apps, VR, neurofeedback, or internet-based interventions.

Regarding its application in mental healthcare in general, research in people with serious mental illness has shown high levels of feasibility and acceptability of games, but there is room for improvement in their design and implementation (Fitzgerald & Ratcliffe, 2020). Few studies have focused on games in forensic settings. One example is the computer-based game StreetWise (Hodge et al., 2015). In this game, users can interact with four different characters in an urban park, who make positive, negative, and neutral suggestions that test the player’s ability and coping skills (Reynolds et al., 2017). Another example is DEEP, a VR-based biofeedback intervention focused on improving diaphragmatic breathing. The better the user breathes, the more progress they make within the virtual underwater world (Bossenbroek et al., 2020; Weerdmeester et al., 2021). While initially developed for adolescents outside of forensic settings, research has shown that DEEP is also very suitable for forensic patients because of its ability to increase engagement, its transdiagnostic focus on diaphragmatic breathing as a coping skill, and its good user experience and ease of use (Bossenbroek et al., 2020; Haneveld et al., 2023).

In general, many games have been developed for adolescents, but little is known about their application in forensic psychiatric youth care. Systematic reviews have shown that games are effective in treatment and prevention of psychiatric disorders in children and adolescents (Wols et al., 2024; Zayeni et al., 2020). While most studies reported significant improvements in symptoms, more robust studies are needed to draw more definitive conclusions.

## Ethical Challenges

In this section, an overview of core ethical points of attention regarding the use of new technology in treatment of forensic psychiatric patients—both youths and adults—is provided. As previously mentioned, this overview is not exhaustive but is intended as a starting point for further research and discussion on ethics and technology in forensic (youth) settings. The ethical points of attention, some of which are related, are illustrated by examples related to the technologies described in the previous sections.

### Informed Consent

An important ethical point of attention regarding the use of interventions in forensic care is informed consent. This refers to patients having sufficient information about the objective and nature of a specific treatment and providing consent to participate on a voluntary basis. Due to the often involuntary treatment context in forensic settings and the dependency patients have on therapists – who control decisions about their leave or discharge – it may be challenging for patients to refuse the use of a technology (Adshead & Davies, 2016; Hempeler et al., 2024; Szmukler & Appelbaum, 2008). Importantly, with respect to unintentional informal coercion, a patient's *perception* of the treatment offered can also be relevant—not just whether there formally is'freedom to choose'(Hempeler et al., 2024; Scholten et al., 2025). In other words, a patient can be completely free to decline an intervention but might unintentionally not perceive the freedom to do so. On the other hand, it is also important that those people in forensic care are offered choices so that choices are not kept away from them for'paternalistic'reasons (Ligthart et al., 2023). Relatedly, it is essential that people in forensic care can also benefit from technological progress, similar to others in (mental) health care. In any case, it is crucial for therapists and/or researchers to clearly communicate that the decision to use a new technology is not related to decisions about a patient’s treatment progress.

Attention to the informed consent procedure is especially relevant for new technologies for which less information is available. A specific group of technologies that serve as an example of this, is neurotechnologies such as tDSC or DBS, which can be viewed as intrusive (Díaz Soto & Borbón, 2022) (see paragraph 2.5). Because little is known yet about their effectiveness and because of the complexity of their working mechanisms, it can be difficult for patients to fully understand the exact nature and implications of innovative treatments, such as VR or tDCS. As described above, many technologies are still'in their infancy', and further research is needed for all classes of technologies discussed, particularly those concerning young forensic patients. In line with this, when seeking informed consent, it is important to prevent patients from having unrealistically positive expectations about the effectiveness of the technology—not only because little is currently known about its impact, but also because existing research shows either no clear evidence of effectiveness or only relatively small effect sizes.

In general, it is important to guarantee that information is fully understood by patients, which is especially relevant for patients with an intellectual disability or lower educational levels, which is relatively prevalent in forensic psychiatric patients (Svensson et al., 2015). This requires tailored communication to individual cognitive skills, with the accompanying pitfall that information is provided in a way that is too complex or unclear for patients (Nishimura et al., 2013). While not much is known yet about shared decision-making, technology and forensic (youth) psychiatry, research in mental healthcare in general shows that it is a valuable tool to better involve people in treatment processes (Francis et al., 2024; Nishimura et al., 2013). A specific point of attention for youths is that, if they are underage, parents or legal guardians must sometimes provide consent for using technologies and thus might have to be involved in the decision-making process (Schneble et al., 2021).

### Privacy and Data Security

Another ethical topic that is highly relevant for working with technologies – particularly those that collect large amounts of potentially sensitive data, which several do – is related to privacy regarding the collection and storage of data that are collected by technologies. This is especially relevant for technologies that are developed by commercial companies, such as consumer wearables or mindfulness apps (Cornet et al., 2017). For example, in the European Union, data are often stored in servers in non-EU countries, such as the U.S., in which legislation concerning data is different from that concerning EU standards. For the patient, it is often not clear who owns their data, who has access, and what is being done with it. For example, companies often have the right to share and sell data, but users do not know exactly which data are being shared or to what extent they are anonymized (Glenn & Monteith, 2014). These are crucial issues for responsible implementation of these technologies by healthcare institutions. However, these matters are also relevant for individual patients. Understanding them can be especially difficult for vulnerable forensic patients with fewer cognitive or digital skills, or for types of data that are harder to understand, such as those collected by neurotechnologies (see paragraph 2.5) (van Dongen et al., 2024). Consequently, when deciding whether to use a technology, issues such as data storage and security must be taken into account, especially with respect to youths, for whom stricter regulations regarding data may exist regarding consent and access to the data (Montgomery & Chester, 2015).

Another point of attention related to legislation is that not all commercial technologies can be used in psychiatry. For example, in the EU, CE certification is required to show that a technology meets legal requirements, including those related to data. If such a certification is not available, it cannot be guaranteed that a technology meets the requirements for use in psychiatric settings (ter Harmsel, 2022). Another topic of attention is the extent to which parents or legal guardians of minors are able and allowed to view data that are collected by technologies. Examples are homework assignments in internet-based interventions or entries in diary or experience sampling apps (see paragraphs 2.2 & 2.3). Ideally, agreements should be made about who can access data, not only parents but also therapists, but there are often no clear guidelines for this. Additionally, clear agreements on who is responsible for possessing knowledge about relevant legislation are important: does this responsibility lie with clinicians, researchers, management, or other professionals?

### Reliability and Validity

An increasing number of technologies in forensic psychiatry collect data and provide feedback to patients based on these data. This raises important questions about the validity and reliability of such data. Validity refers to the extent to which a technology accurately measures what it is intended to measure, whereas reliability refers to the consistency and stability of the measurements over time.

An example of this are wearables that provide biofeedback based on physiological data such as heart rate, steps per day, or sleep duration (see paragraph 2.4). While these physiological signals can be measured reliably, their validity as indicators of psychological states—such as stress or anxiety—is more problematic (Hickey et al., 2021). A raised heart rate, for instance, may reflect physical exertion, excitement, or stress, making it an ambiguous marker of mental state. A pitfall of this is that patients might *believe the device* rather than their own assessment. In some cases, such feedback can increase anxiety or undermine emotional self-awareness. In general, it is important to prevent technologies from prescribing patients how they feel, which hinders their autonomy and can decrease their—often already low—emotional awareness.

Another example involves experience sampling apps, which are used to assess patients’ daily mood, aggression, or stress in real time (see paragraph 2.3). These tools aim to provide personalized feedback or data for use in treatment. However, assessing the validity and reliability of experience sampling data is challenging (Stone et al., 2023). Self-reports can vary depending on how patients interpret the questions, their level of literacy or cognitive ability, and their capacity for introspection. For instance, individuals with low interoceptive awareness may struggle to accurately report on internal states like stress, regardless of how frequently or consistently they are prompted, or patients might not fully understand the questions that are asked due to complex formulation (Heirbaut et al., 2024). This variability can compromise both the reliability (e.g., inconsistent responses over time) and the validity (e.g., inaccurate reflection of true experience) of the data collected.

A way to (partially) overcome this is by providing explanation and supervision to therapists to ensure that they have a good understanding of the limitations of a technology (Heirbaut et al., 2024). However, therapists often indicate that they do not have enough knowledge, skills or time to thoroughly introduce, supervise and integrate these technologies in treatment (Bosch et al., 2024; Heirbaut et al., 2024).

### Equity, Accessibility, and Usability

An increasing body of research shows that technology has the potential to improve treatment of forensic (youth) patients. However, at this point in time, not all patients benefit equally from the advantages of these new interventions. A barrier is related to accessibility: not all possess technologies that are necessary to receive interventions, such as smartphones or smartwatches. A related issue arises when using technology in closed settings, in which inpatients are often not allowed to use technologies with internet access, such as smartphones. Another topic of attention regarding this ‘digital divide’ is related to digital skills: not all patients have sufficient skills to independently use technologies. An example of this can be found in a usability study on a mobile diary app, in which some patients indicated that navigating through the app was too difficult for them, causing them to feel ‘not smart’ and that the app was obviously made by people ‘smarter than them’ (Dekkers et al., 2024). For most youths, who are raised with technology and are digital natives, this issue seems to be less relevant. However, owing to their abundant experience with commercially developed technology, youths might have high expectations of the user experience, which can cause them to stop using technological intervention earlier because their design does not meet their expectations.

Another topic of attention is the fit between the content of a technology and a patient’s cognitive skills. Many technologies, such as internet-based interventions (see Paragraph 2.2) and some types of mobile apps (see Paragraph 2.3), contain much written language and assignments that require a high level of cognitive reflection (Kip et al., 2020). Additionally, wearables with biofeedback (see Paragraph 2.4) or experience sampling apps often contain visualizations of collected data that require interpretation. However, as mentioned before, a relatively high number of forensic (youth) patients have intellectual disabilities or other types of cognitive impairments that might make independent use of some technologies too challenging. A related point is that therapists sometimes decide for patients whether they are able to use a technology, for example, because they expect that a patient will not be able to understand the technology and, in that way, deny them access to a technology that could have potentially been of added value (Kip et al., 2020). In general, technology is framed as a tool to decrease disparities between people from different backgrounds. While technology can contribute to increasing access to healthcare for vulnerable populations, in practice, the opposite often occurs (Olfson et al., 2025). Many technologies are more accessible to those with more technological and literary skills, such as highly educated females,. Therefore, instead of decreasing healthcare disparities, the use of technology could contribute to its further increase.

An issue related to accessibility is that many organizations and therapists do not offer the same level of access to technologies. In other words, while patients might be able to use internet-based interventions in one organization, this might not be an option for patients who are treated by another organization (Bierbooms et al., 2015). This even differs within a clinic: while one therapist is able and willing to offer treatment with VR or wearables, another therapist might not be able or willing to offer this option (Feijt et al., 2018). A consequence of all of these aforementioned barriers is that, in practice, a large percentage of forensic patients are not able to access and use potentially effective interventions.

### Undesired Side Effects

Technology may have unintended effects. One example concerns strong emotions. Examples are patients who are immersed in VR scenarios that trigger overwhelming emotions (see paragraph 2.1), users who are in a state of flow within a game (see paragraph 2.6), or wearables that provide confronting feedback about stress levels (see paragraph 2.4). Ideally, these emotions are sufficiently addressed during treatment and serve therapeutic purposes. However, there is a risk of eliciting unwanted emotions without having enough possibilities to reduce them (Cornet & Van Gelder, 2020; Kip et al., 2024). This can happen outside of treatment settings – think of a reflective assignment in an internet-based intervention that causes a patient to spiral without a therapist being present to help them deal with it. It can also occur during treatment, for example, when craving is elicited in VR without sufficient reduction, causing the patient to buy drugs directly after leaving the treatment room. This can also happen unintentionally: think of accidentally exposing patients to triggers of underlying traumas that are not known to the therapist in VR. Because therapists have more control over the shape and content of VR scenarios, this might also imply that they have more responsibility regarding the elicitation and regulation of intense emotions that patients experience because of the use of these technologies. Clearly, discussing the content of a technology beforehand when patients are obtained—and in the case of minors, their parents'or legal guardians'—consent is a way of reducing this risk.

### Acceptability of Content

A theme that is specifically relevant for forensic care – with its focus on delinquent behaviours – is related to the extent to which content and images in technologies are acceptable (Kip et al., 2024). This is especially relevant for technologies that rely on visual cues such as the use of virtual naked children in assessment of paedophilia VR (see paragraph 2.1). In addition to the legal aspect regarding the extent to which this can be viewed as generating virtual child porn, this approach can also inspire ethical discussions about the desirability of exposing such sensitive images to patients and the emotions they can elicit (Trottier et al., 2019). Another topic related to the treatment of forensic patients involves questions about how far a therapist can go in attempting to provoke or challenge the patient within VR scenarios. In line with this, which aggressive or sexually deviant reactions from patients can and cannot be accepted in virtual environments (Kip et al., 2024)? In other words, this comes down to establishing the ethical boundaries of (interactions in) the virtual world in forensic psychiatry, particularly where it concerns vulnerable youths.

Acceptability of content is also related to equity and diversity in the design of technology. It is important to prevent stigma when developing materials such as avatars, videos, or pictures. On the one hand, subgroups might feel underrepresented in a technology, e.g., when only videos of people of one ethnicity or gender are shown in internet-based interventions. On the other hand, people with a specific ethnic background might feel stigmatized because people with a similar background are reflected in a negative way in VR scenarios, e.g., in aggressive behaviour (Klein Schaarsberg et al., 2024; Ligthart et al., 2022a, 2022b).

### Persuasiveness

An advantage of many technologies is that they are persuasive. In other words, they are designed to support people in changing their attitudes and behaviours (Oinas-Kukkonen & Harjumaa, 2009). Persuasive features can be added to technologies to increase user adherence, engagement and effectiveness. Examples include reminders to complete assignments in mobile apps, personalization of VR scenarios, rewards when reaching a certain objective (e.g., steps per day) with a wearable, competition in games, or adding endorsement and authority to internet-based interventions by adding logos from trusted organizations (Oinas-Kukkonen & Harjumaa, 2009). Persuasive systems can also raise ethical questions, especially if they are not performed well (Kellmeyer, 2018; Kellmeyer et al., 2019; Ligthart et al., 2022a, 2022b). Unnecessary stress can arise if a patient does not follow a persuasive suggestion from an app or internet-based intervention (Lupton, 2012).

An important characteristic of persuasive technologies is their ability to monitor and influence behaviour continuously, 24/7, 7 days a week. Examples are wearables that continuously monitor and provide feedback on stress-related variables (see paragraph 2.4) or experience sampling apps that ask the user throughout the day how they are feeling and provide suggestions for improvement on the basis of their input (see paragraph 2.3). The technology is always available, possibly reminding patients of treatment and sensitive topics, especially during moments in which this is not desirable. These persuasive features of technologies can be experienced as intrusive, patronizing or an invasion of autonomy (ter Harmsel et al., 2023a, 2023b). Unlike a human persuader, a technology cannot estimate whether persuasive suggestions become too intense or intrusive (Fogg, 2002). For example, a biosensor does not feel guilty but keeps on sending notifications as long as it is being used. In the design of these technologies, emphasis should be placed on the potential downsides of persuasiveness, and strong arguments should be made for the use and timing of persuasive elements (e.g., are reminders always necessary, how many times a day should they be sent, can users stop the reminders, etc.).

### Evidence-based Interventions

In general, the use of evidence-based interventions is considered important in forensic psychiatry. However, as shown by the overview of technological interventions in Sect."Opportunities of Technology for Forensic Psychiatric Youth Care", few robust effectiveness studies exist on technology in treatment of forensic patients, and even less is known about its added value for treatment in forensic psychiatric youth care. Consequently, institutions such as healthcare providers, governments and forensic organizations may encourage the use of technologies that are not sufficiently studied or evidence-based. Additionally, hardly anything is known about which intervention works for which patient, making it difficult to draft indication criteria and use technologies in a targeted way (Kip et al., 2019a, 2019b, 2019c). This raises questions about why technologies are used: are they the best option to increase treatment quality for individual patients, or is their introduction driven mostly by a need to innovate for the sake of innovating? In general, it is important to carefully discuss the amount of evidence that is required before introducing a new technological intervention in treatment of forensic patients. Many technologies are quite costly, e.g., some VR systems or subscriptions to platforms that offer internet-based interventions, and implementation requires much time and effort from therapists and other employees. To what extent is it justified to invest in interventions whose effectiveness requires more evidence?

## Ways Forward

In the previous section, we considered several ethical questions related to the use of different types of technology in forensic psychiatry, with a focus on treatment of youths. Based on research on virtual reality, internet-based interventions, mobile apps, wearables with biofeedback, neurotechnology, and games in forensic settings, we highlighted multiple ethical points of attention related to informed consent, reliability and validity, privacy and data security, equity and accessibility, undesired side effects, acceptability of content, persuasiveness, and evidence-based interventions. It is important to carefully consider these types of questions, not only because the use of technology as a treatment tool in forensic settings is relatively new but also because technology has unique characteristics compared with humans, such as immersiveness, 24/7 presence, and persuasiveness. Furthermore, the rapid pace of technological development can hinder careful consideration of potential ethical challenges in the short and long term. Additionally, forensic patients—and more specifically, youths—are an especially vulnerable group. The unique characteristics of both technology and forensic psychiatric youth care make it especially important to pay more attention to ethics.

In our view, the ethical issues that were mentioned do not allow for'quick fixes'. As a starting point, mere awareness of these concerns is valuable. In the next step, we may ask ourselves how to address the problems that were identified. The points that were raised in this paper could serve as the basis for guidelines to discuss ethical issues when developing, implementing, and evaluating technology in forensic psychiatric (youth) settings. In what follows, we suggest ways forward for the responsible use of technologies in forensic psychiatric youth care. They are meant to be applicable across a range of technologies and a range of ethical questions: the ones mentioned in this paper, but also new ones that may emerge due to new developments and insights.

### The Importance of Co-creation

A valuable way to address ethical matters in a timely and careful manner is by incorporating a broad range of perspectives in an interdisciplinary team that coordinates the development and implementation of technology (Ligthart et al., 2022a, 2022b; Scholten & Granic, 2019). Such a team should involve not only developers, ethicists, managers, and researchers from different backgrounds but also forensic psychiatric patients and therapists. Ideally, their input should be gathered throughout every step of the process (Kip et al., 2019a, 2019b, 2019c). This means including them in decision-making from the very beginning of the development of a new intervention to its implementation and evaluation in practice. An approach to achieve this is co-creation, also referred to as participatory development. In such an approach, stakeholders such as patients, caregivers, managers, researchers, technology developers, ethicists, and legal experts are involved from the beginning in creating or improving a technology (Dekkers & Burns, 2024). By actively involving stakeholders, their values, wishes, needs, and ideas regarding the technology are considered from the beginning. This approach not only ensures that a technology matches the values of end users but also prevents ethical issues from becoming apparent only after the introduction of an intervention in practice (Klein Schaarsberg et al., 2023; Klein Schaarsberg et al., 2024; Van de Poel & Royakkers, 2023; van Gemert-Pijnen et al., 2011).

Despite its recognized importance for developing technologies that fit the people and contexts involved, the active involvement of patients in co-creation is not common practice in forensic settings or even in mental healthcare in general (Kip et al., 2019a, 2019b, 2019c; Schouten et al., 2022). Additionally, if co-creation is applied, patients may not be seen as equal partners in the process but more as passive informants who are not involved from the start (e.g., during the brainstorming phases of a project), merely providing feedback on already developed interventions, resulting in less opportunity to provide input on, e.g., the overall objective and content of an intervention (DeSmet et al., 2016).

Thoroughly applying co-creation entails that developers truly listen to the input of patients, even if it is not in line with initial ideas or beliefs (Dekkers & Burns, 2024). Importantly, user input is not always central: there remains a delicate balance between the theoretical foundation of an intervention and the input of prospective end-users and other stakeholders. When combining research, practice, and end-user perspectives, there might be conflicting values—think of patients (or other stakeholders) who suggest options that are not in line with legislation. One way to address this is by ensuring that all decisions about an intervention are made by an interdisciplinary team that contains different perspectives: not only researchers and developers but also patients and therapists, who collaborate as equal partners (Schouten et al., 2022). It is important to note that such an approach towards co-creation is time intensive and might require an attitude shift of researchers and developers (Schouten et al., 2022). However, we believe that this process, in the end, might also be'cost-effective', as the eventual implementation of these technologies may benefit from such a careful development procedure. Finally, even if co-creation methods are used, thorough attention to ethics may still be lacking. An important recommendation is to involve ethicists in interdisciplinary development teams to ensure that there is a sufficient focus on ethics throughout the entire process (Scholten & Granic, 2019). Development of ethical guidelines – partly based on this perspective paper – is recommended to support this process.

### Evaluation Studies to Gain Insight into what Works for Whom

As has become clear from the overview of studies presented in this paper, not much is known yet about which technological intervention works best for which type of patient, in which type of treatment and context. From an ethical point of view, offering interventions that have been proven to be effective is important, and providing enough information about if, how and for whom an intervention works is of added value when asking for informed consent. An obvious recommendation is to conduct more thorough evaluation studies that focus not only on if but also on why, for whom, and when interventions are effective. Throughout the years, it has become increasingly clear that RCTs are often not the golden standard for the evaluation of technological interventions, not only because technologies can change rapidly and RCTs take up much time but also because RCTs do not provide enough insight into *why and how* an intervention works or how to optimize it (Bonten et al., 2020; Sieverink et al., 2018). Indeed, studies note that not all technologies work for all patients. This shows that a one-size-fits-all approach towards technology is not suitable, especially for heterogeneous forensic populations that require responsive, personalized treatment (Grove et al., 2021; Kip et al., 2018). Consequently, iterative, multi-method evaluation processes in which patients and therapists are actively involved via, e.g., qualitative research or participation in research teams have been recommended (Kip et al., 2019a, 2019b, 2019c). Methods such as single case (experimental) designs, mixed-method pilots, or fractional factorial designs can contribute to opening the black box of technological interventions in forensic (youth) care. An accompanying advantage of this iterative, multi-method approach is that there is more room for attention to ethics throughout the evaluation process, mostly because the perspective of end-users is continuously taken into account.

### Shared Decision-making for Integration in Treatment

A consequence of the need for more insight into effectiveness and working mechanisms of technology in forensic psychiatry, is that it is difficult, or even impossible, to make evidence-based decisions about which technology to use for which patient. This does not mean that technology can or should not be used: it merely requires more careful deliberation. Shared decision-making about treatment options can better involve patients in the process of deciding which form of treatment – in this case, technology—would be the best fit for their individual situation (Francis et al., 2024). Actively involving them in deciding which technology to use and determining how to integrate this technology in their treatment can overcome at least some of the ethical challenges described in this paper (Francis et al., 2024). An important aspect of this, especially when treatment is obligatory, is clear and transparent communication about the possibility to refuse an intervention (Hempeler et al., 2024). Based on other research on shared decision-making in mental healthcare in general, we expect that putting patients more central in decision-making about technology will result in patients who are better informed, have more ownership, and receive more opportunities to have a stake in the decision to start or stop using a technology (Hamann & Heres, 2014; Nishimura et al., 2013).

A necessary precondition for shared decision-making is that therapists have sufficient knowledge about different technologies that can be used and their potential positive and negative consequences. However, to our knowledge, this is often not the case: therapists rarely receive any training in the use of technology. If training is provided, it is often focused on only one technology, and it often emphasizes only technical skills, overlooking important topics such as relevant therapeutic skills, integration in treatment, and, of course, ethical topics of attention (Kip et al., 2020; Kouijzer et al., 2023). To overcome this, more attention needs to be paid to implementation. This requires not only attention to therapist training but also changes within forensic organizations, the design of a technology, attention to patient preferences, and national legislation and policy (Greenhalgh et al., 2017).

## Limitations

In line with the characteristics of viewpoint papers and ethical research, this paper was created through a general examination of the relevant literature. Given the nature of this article, no systematic literature search was conducted. As mentioned above, the issues raised should therefore not be seen as an exhaustive overview but rather as initial directions for further research and practical reflection. Nevertheless, the overview of technologies and ethical points of attention is based on several systematic reviews on technology in treatment of forensic youths and people who committed an offense, and when describing the effectiveness of technologies, systematic reviews and meta-analyses are used as much as possible. Furthermore, this article focuses on six types of technologies, but many of the highlighted points are also relevant for other types of technologies, such as videoconferencing or social media platforms. Another limitation is the lack of research specifically focused on forensic psychiatric youth care. As a result, most of the sources used pertain to adults receiving forensic psychiatric treatment. Although there are many similarities between these groups, certain ethical considerations unique to youths may be overlooked due to the relative scarcity of research on this topic. Finally, due to the rapid developments in the field of technology—e.g., the quick rise of artificial intelligence—new ethical points of attention are likely to arise, which shows that the overview in this paper is not exhaustive but is meant as a starting point for further discussion.

## Conclusion

Based on the overview of technologies in this viewpoint paper, it can be concluded that not much is known yet about the use of technology in forensic psychiatric youth care. This does not just refer to insights into if, why, and for whom a technology works but also to ethics. Due to the rapid rise of new and interesting technologies, it may be easy to overlook ethical points of attention. However, especially when working with complex and vulnerable populations, such as patients in forensic psychiatric youth care, it is important to carefully consider these ethical aspects. A way to facilitate this is by actively involving patients and therapists throughout all phases of the process: development of the technology via co-creation, active participation in its implementation in practice, and accounting for their perspectives during evaluation. Inter- or even transdisciplinary collaboration between patients, therapists, technology developers, forensic organizations, ethicists and researchers from backgrounds such as psychology, ethics, law, and design sciences will contribute to a more timely identification of ethical challenges and the creation of suitable solutions. In our view, if designed and used well – taking into account ethical concerns – new technologies clearly have the potential to improve forensic psychiatric treatment of youths.

## Data Availability

Not applicable.
